# Predictors of treatment dropout in patients with posttraumatic stress disorder due to childhood abuse^1^

**DOI:** 10.3389/fpsyt.2023.1194669

**Published:** 2023-08-04

**Authors:** Susanne Bremer-Hoeve, Noortje I. van Vliet, Suzanne C. van Bronswijk, Rafaele J.C. Huntjens, Ad de Jongh, Maarten K. van Dijk

**Affiliations:** ^1^Dimence Mental Health Group, Deventer, Netherlands; ^2^Department of Psychiatry and Neuropsychology, School for Mental health and Neuroscience, Faculty of Health Medicine and Life Sciences, Maastricht University, Maastricht, Netherlands; ^3^Department of Psychiatry and Psychology, Maastricht University Medical Center, Maastricht, Netherlands; ^4^Department of Experimental Psychotherapy and Psychopathology, University of Groningen, Groningen, Netherlands; ^5^Department of Social Dentistry and Behavioral Sciences, University of Amsterdam and Vrije Universiteit, Amsterdam, Netherlands; ^6^School of Health Sciences, Salford University, Manchester, United Kingdom; ^7^Institute of Health and Society, University of Worcester, Worcester, United Kingdom

**Keywords:** PTSD, childhood abuse, EMDR, dropout, predictors, machine learning

## Abstract

**Background:**

Knowledge about patient characteristics predicting treatment dropout for post-traumatic stress disorder (PTSD) is scarce, whereas more understanding about this topic may give direction to address this important issue.

**Method:**

Data were obtained from a randomized controlled trial in which a phase-based treatment condition (Eye Movement Desensitization and Reprocessing [EMDR] therapy preceded by Skills Training in Affect and Interpersonal Regulation [STAIR]; *n* = 57) was compared with a direct trauma-focused treatment (EMDR therapy only; *n* = 64) in people with a PTSD due to childhood abuse. All pre-treatment variables included in the trial were examined as possible predictors for dropout using machine learning techniques.

**Results:**

For the dropout prediction, a model was developed using Elastic Net Regularization. The ENR model correctly predicted dropout in 81.6% of all individuals. Males, with a low education level, suicidal thoughts, problems in emotion regulation, high levels of general psychopathology and not using benzodiazepine medication at screening proved to have higher scores on dropout.

**Conclusion:**

Our results provide directions for the development of future programs in addition to PTSD treatment or for the adaptation of current treatments, aiming to reduce treatment dropout among patients with PTSD due to childhood abuse.

## 1. Introduction

Posttraumatic stress disorder (PTSD) has a major impact on social and occupational functioning and individuals’ quality of life (1). In addition, individuals with PTSD are at great risk of attempting suicide (2–4). Although many treatments targeting PTSD have proven to be effective [e.g., (5)], a recent meta-analysis of randomized controlled trials (RCTs) found an average dropout rate of 21% for guideline-recommended PTSD treatments (6). This is problematic as untreated PTSD compared to treated PTSD may lead to a worse prognosis, and many societal consequences (7). Considering the societal impact of PTSD, it is important to ensure that individuals suffering from PTSD complete their treatment because evidence-based PTSD treatments significantly improve their prognosis (7). Completing treatment may be of particular importance for those with a history of childhood abuse since they are at risk of displaying more severe symptoms of PTSD or developing symptoms of Complex PTSD [ISTSS, 2012; (8)].

In order to establish a Complex PTSD diagnosis individuals need to experience symptoms of “Disturbances in Self-Organization” [DSO; i.e., problems with affect regulation, negative self-concept, and interpersonal problems; (9)], in addition to all diagnostic criteria of PTSD. It has been suggested that existing evidence-based trauma-focused therapies may lead to less favorable outcomes and more dropout in patients with a history of childhood abuse (10–12), and that these patients are at risk of developing Complex PTSD (8). Previous attempts to decrease the dropout rate from PTSD treatments in this target group by adding treatment programs that specifically target Complex PTSD symptoms in addition to actual trauma-focused treatment (11, 13) did not lead to less dropout compared to only applying direct trauma-focused treatment (14–16). The identification of patient characteristics that predict early treatment termination is essential for developing new target strategies to prevent dropout.

Most meta-analyses studying potential predictors of dropout regarding PTSD treatments focus on the kind of therapy as a predictive factor [i.e., (17–21)]. As far as we know, only Varker et al. (6) performed a meta-analysis in which they also considered patient characteristics in the comparison of several PTSD treatments among patients with military and civilian trauma. PTSD chronicity, PTSD severity, medication use, age, employment status, relationship status, sex, baseline depression scores, or baseline anxiety scores were included separately as possible predictors, but none of the included patient characteristics were found to be predictive of dropout. To this end, given the scarcity of studies and variables considered so far and the lack of meta-analytic support for specific comorbid symptoms as potential predictors for treatment dropout, more research on patient characteristics predicting dropout is needed. As, until now, not one specific characteristic has been found to be predictive of dropout, it may be even more important to determine whether a combination of characteristics may be predictive.

The purpose of the present study was to identify patients who are at risk of dropping out, and to identify patient characteristics that predict the dropout of PTSD treatments in patients with PTSD related to childhood abuse. To achieve this aim, we used machine learning techniques. One advantage of machine learning techniques is that all available pretreatment variables and variable combinations are examined (22), thus not limiting the number of possible predictors. Another advantage is that it is particularly appropriate for identifying small effects in predicting outcomes (22). For the current study, the dataset of a multi-center randomized controlled trial was used, that compared a phase-based treatment with a direct-trauma-focused treatment in patients with PTSD related to a history of childhood abuse [Van Vliet et al., 2017; (16)].

## 2. Method

### 2.1. Design and participants

A total of 121 patients were recruited in two mental health organizations; Dimence GGZ and GGZ Oost-Brabant. Included patients were aged between 18 and 65 years, and diagnosed with PTSD based on the Clinician-Administered PTSD scale for DSM-5 [CAPS-5; (23)]. Participants had to be a victim of repeated sexual and/or physical abuse before the age of 18 by a caretaker or a person in a position of authority, which was identified by the Life Events Checklist for the DSM-5 [LEC-5; (23)]. Patients were excluded when they did not master the Dutch language sufficiently, or in case of acute suicidality for which direct crisis intervention was needed, as assessed by an item of the Beck Depression Inventory-II [BDI; (24)]. In addition, patients were excluded if they had received at least eight sessions of any well-evaluated treatment for PTSD in the past year, reported being a victim of ongoing physical and/or sexual abuse, reported severe use of alcohol or drugs, or had an intellectual disability. The study design was registered in a national trial registry^2^ and approved by the medical ethics committee Twente NL.^3^ Further details on the trial methodology and patient sample can be found in the study protocol (25) and main outcome paper of the study (16).

### 2.2. Interventions

After patients were found to be eligible for participation in the study, they were randomized to two treatment conditions: both contained 16 sessions of EMDR (Eye Movement Desensitization and Reprocessing) as the trauma-focused treatment, and in one condition these sixteen sessions of EMDR were preceded by eight sessions of the first phase treatment STAIR (in total 24 sessions). Both the STAIR and the EMDR therapy were delivered twice a week for 90 min. STAIR was conducted according to the program described by Cloitre and her colleagues (11). EMDR therapy was conducted according to the protocol by Shapiro using the Dutch translation of the treatment protocol (26). Before actual treatment, patients first received one 90 min session consisting of psycho-education in which a hierarchy of relevant traumatic experiences was determined. For a full description of the two treatments, see Van Vliet et al. (25).

### 2.3. Measures

#### 2.3.1. Drop out

Patients were considered to have dropped out if they discontinued treatment prematurely after the first session, which included psychoeducation and case conceptualization just before the actual treatment began. This applies to cases where the patient did not complete the total number of treatment sessions as per the study’s requirements [see the study protocol; (25)], and failed to complete treatment for all the traumas that were selected for treatment during the first treatment session. The outcome variable was a dichotomous variable: dropout versus completer status.

#### 2.3.2. Pre-treatment variables

Tables 1 and 2 show an overview of the pre-treatment variables and the differences between the two groups (dropout versus completer status). The reasons given by the patients for dropout are shown in Supplementary Table S1.

**Table 1:** Continuous baseline variables pre-treatment for completers and dropouts and comparison of the means.

Variables pre-treatment	Completer (*n* = 90) Mean (*Sd*)	Dropout (*n* = 21) Mean (*Sd*)	*t*	df	*p*
Age	37.91 (12.72)	34.59 (10.57)	1.11	109	0.27
	38.32 (9.27)	39.57 (8.27)			
CAPS-5	31.28 (11.73)	34.19 (14.31)	−0.57	109	0.57
	29.03 (9.02)	31.19 (7.09)			
SIDES	23.55 (14.82)	26.06 (14.64)	−0.98	109	0.33
	112.79 (24.87)	120.52 (22.15)			
PSS-SR	3.98 (1.40)	4.29 (1.07)	−1.03	109	0.31
	1.63 (0.57)	1.71 (0.53)			
DES	1.83 (0.72)	2.09 (0.75)	−0.7	109	0.49
	37.91 (12.72)	34.59 (10.57)			
DERS	38.32 (9.27)	39.57 (8.27)	−1.31	109	0.19
	31.28 (11.73)	34.19 (14.31)			
PTCI Self-Esteem	29.03 (9.02)	31.19 (7.09)	−0.96	109	0.34
	23.55 (14.82)	26.06 (14.64)			
IIP	112.79 (24.87)	120.52 (22.15)	−0.59	109	0.56
	3.98 (1.40)	4.29 (1.07)			
BSI	1.63 (0.57)	1.71 (0.53)	−1.47	109	0.14
	1.83 (0.72)	2.09 (0.75)			

CAPS-5, Clinician Administered PTSD Scale for DSM-5, SIDES = Structured Interview for Disorders of Extreme Stress-Revised, PSS-SR, PTSD Symptoms Scale-self report, DES, Dissociative Experiences Scale, DERS, Difficulties in Emotion Regulation Scale, PTCI, Posttraumatic Cognitions Inventory, IIP, Inventory of Interpersonal Problems, BSI, Brief Symptom Inventory.

**Table 2:** Categorical baseline variables for completers and droupouts and comparison of the amounts.

Variable	Completer (*n* = 90)	Dropout (*n* = 21)	*ꭓ^2^* / Fisher’s exact	*df*	*p*
Gender					
Woman	65	11	2.25	1	0.13
Man	25	10			
Education					
low	42	12	4.06	2	0.13
middle	33	9			
high	15	0			
Employment					
unemployed	54	14	0.33	2	0.85
employed	25	5			
student	11	2			
Living together	53	12	0.00	1	1.00
Married	40	8	0.08	1	0.78
Sexual Abuse	69	14	0.45	1	0.50
Physical Abuse	69	16	0.00	1	1.00
Dissociative subtype	31	6	0.66	1	0.80
Complex PTSD	22	10	3.40	1	0.07
Borderline personality disorder	16	5	0.11	1	0.74
Self-injury					
no self-injury	53	12	0.09	2	0.96
doubtful	14	3			
self-injury	23	6			
Suicidality			5.75	1	0.02^*^
no suicidality	27	1			
suicidality	63	20			
Psychiatric medication use	48	13	0.22	1	0.64
Benzodiazepine medication use at screening	22	2	2.24	1	0.24

*p < 0.05.

##### 2.3.2.1. Demographic characteristics

The following demographic characteristics were determined in the study: Gender, level of education, employment status, marital status, and age.

##### 2.3.2.2. PTSD variables

PTSD symptom severity at the start of the treatment was measured with the CAPS-5 (23). The CAPS-5 is a clinical interview that includes 20 items on a 5-point Likert scale, resulting in a total score of between 0 and 80. The CAPS-5 has good psychometric properties [(27); see for the Dutch version (28)]. The inter-rater reliability was assessed by calculating the interclass correlation coefficient, which was 0.999, which is an excellent score.

##### 2.3.2.3. Suicidality

Item 9 of the BDI-II (24) was used to measure suicidality. The scale of this item ranges from 0 to 3, with 0 for the absence of suicidal thoughts, 1 for indicating the presence of suicidal thoughts, but no intention to carry them out, and, 2 for indicating suicidal thoughts accompanied by a clear intention to commit suicide. Patients were excluded when they scored a 3 on this scale, which said that they would commit suicide whenever they had the chance. In that case they were assigned to a direct crisis intervention. In the current analysis, suicidality was used as a dichotomous value: absence (score of 0) or presence of suicidality (score of 1 or 2).

##### 2.3.2.4. Borderline personality disorder

The Structured Clinical Interview for DSM-IV Axis II personality disorders [SCID-II interview; (29, 30)] was used to determine the presence of a borderline personality disorder. The psychometric properties are fair to good for this instrument.

Self-Injury: The severity of self-injury was determined by items 97 and 98 of the Dutch version of the SCID-II interview (30), with 1 for absent, 2 for doubtful, and 3 for present.

##### 2.3.2.5. Dissociative symptoms

The severity of dissociative symptoms was indexed using the Dissociative Experiences Scale (DES-II; (31); Cronbach’s *α* = 0.93 in the present study at baseline). This is a 28-item self-report questionnaire with scores ranging from 0 to 100 (32). The presence of a dissociative subtype of PTSD was determined using the CAPS-5 (23).

##### 2.3.2.6. Complex PTSD

The presence and severity of Complex PTSD was measured by the Structured Interview for Disorders of Extreme Stress [SIDES; (33)], the 38-item version developed by Ford et al. (34). The SIDES has good psychometric properties as a dichotomous measure in determining whether Complex PTSD is present or not [SIDES Manual by (35)].

##### 2.3.2.7. Interpersonal difficulties

Interpersonal difficulties were indexed using the Inventory of Interpersonal Problems [IIP; (36)]. The psychometric properties of the IIP are satisfying (37). The IIP contains 32 items that can be scored on a 5-point scale from 0 (not at all) to 4 (very strongly). The reliability at baseline in this study was high (Cronbach’s *α =* 0.85).

##### 2.3.2.8. Emotion regulation

Difficulties in emotion regulation were measured with the Difficulties in Emotion Regulation Scale [DERS; (38)], a questionnaire that has been validated in clinical populations (38, 39) and nonclinical populations (38, 40). Each item of the DERS is rated on a 5-point scale. The reliability in this study at baseline was high (Cronbach’s *α* = 0.92).

##### 2.3.2.9. Problems In self-esteem

To index problems in self-esteem the self-esteem subscale of the Posttraumatic Cognitions Inventory [PTCI; (41)] was used. Items are scored on a Likert scale from 1 (“I totally disagree”) to 7 (“I totally agree”), and psychometric properties for the Dutch version (42) are good. The PTCI score for self-esteem showed a high reliability at baseline in this study (Cronbach’s *α* = 0.94).

##### 2.3.2.10. General psychopathology

The Brief Symptom Inventory (43, 44) was used to measure the severity of general psychopathology symptoms. The severity of each item can be rated on a 5-point scale from 0 (not at all) to 4 (a lot). The Dutch version has good psychometric properties [(45); Cronbach’s *α* = 0.95 at baseline in the present study].

## 3. Statistical analysis

All analyses were carried out using RStudio (46). The R code used is available in the Supplementary Material.

### 3.1. Data pre-processing

Following Cohen et al. (47) individuals with less than 50% missing pre-treatment values were included in this study. As a result, ten individuals were excluded. For the remaining 111 participants, variables with missing data were imputed using a random forest imputation algorithm [R package ‘MissForest’, (48)]. The benefits of this approach are that no pre-processing is required and that it is robust for noisy data and multicollinearity, so that it can be applied to mixed data types (48, 49). The imputation method was verified by applying this method to the non-missing dataset with completely at random removed values. After imputing the missing values, the performance was assessed using the normalized root mean squared error (NRMSE) and the proportion of falsely classified (PFC), which is defined by comparing the complete values with the imputed values (48).

In case of highly correlated variables (cor. > 0.70), one of the variables was dropped to avoid redundancy and multicollinearity. The decision which variable to remove was made by the research team. Outliers were winsorized and continuous variables with a non-normal distribution were transformed using the Box-Cox method [(50); R package ‘Caret’; (51)]. Finally, continuous variables were standardized and categorical variables were centered (see Table 3 for details about transformations for each variable). This data pre-processing procedure resulted in a dataset of 111 participants with 22 pre-treatment variables. Dropouts did not differ significantly between patients who received STAIR-EMDR (21.8%) and EMDR only therapy [16.1%; ꭓ2 (1) = 6.00, *p* = 0.440]. A prognostic model for dropout was developed independently of treatment conditions because the subsample of dropouts was too small to create a prescriptive model for dropout depending on the treatment conditions.

**Table 3:** Variable transformation.

Variable	Included	Reason excluded	Transformation
Sex	yes		Centered (male: −0.5, female: 0.5)
Education	yes		Centered (low: −0.5, middle: 0; high: 0.5)
Employment	yes		Centered (unemployed: −0.5, student: 0; employed: 0.5)
Age	yes		Transformed (lambda 0.4)
Married	yes		Centered (not married: −0.5, married: 0.5)
Living together	no	Substantial overlap with variable Married	
LEC-5 Sexual Abuse	yes		Centered (no sexual abuse: −0.5, sexual abuse: 0.5)
LEC-5 Physical Abuse	yes		Centered (no physical abuse: −0.5, physical abuse: 0.5)
Dissociative subtype	yes		Centered (no dissociative subtype: −0.5, dissociative subtype: 0.5)
BDI-II item 9 Suicidal thoughts	yes		Centered (no suicidal thoughts: −0.5, suicidal thoughts: 0.5)
Complex posttraumatic stress disorder (PTSD)_	yes		Centered (no complex PTSD: −0.5, complex PTSD: 0.5)
SCID-II item 97 and 98 Borderline personality disorder (BPD)	yes		Centered (no BPD: −0.5, BPD: 0.5)
Kind of Personality Disorder	no	Substantial overlap with variable Presence BPD	
Self-injury	yes		Centered (no self-injury: −0.5, doubtful: 0; self-injury: 0.5)
Posttraumatic stress disorder (PTSD)_	no	Near-zero variance	
Psychoactive medication use at screening	yes		Centered (no Psychoactive medication: −0.5, Psychoactive medication: 0.5)
Benzodiazepine medication use at screening	yes		Centered (no Benzodiazepine medication: −0.5, Benzodiazepine medication: 0.5)
CAPS-5 total score	yes		
SIDES total score	yes		
PSS-SR total score	yes		
DES total score	yes		Winsorized 2 high outliers; Transformed (lambda 0.4)
BSI total score	yes		
PTCI total score	no	Subscale included	
PTCI Self-Esteem	yes		Transformed (lambda 1.2)
DERS total score	yes		Transformed (lambda 1.5)
IIP total score	yes		

Transformations were performed with the BoxCox method.

BDI-II, Beck Depression Inventory; CAPS-5, Clinician-Administered PTSD scale for DSM-5; SIDES, Structured Interview for Disorders of Extreme Stress; PSS-SR, PTSD Symptoms Scale-Self Report; DES-II, Dissociative Experiences Scale; BSI, Brief Symptom Inventory; PTCI, Posttraumatic Cognitions Inventory; DERS, Difficulties in Emotion Regulation Scale; IIP, Inventory of Interpersonal Problems; SCID-II, Structured Clinical Interview for DSM-IV Axis II; LEC-5, Life Events Checklist for DSM-5.

### 3.2. Imbalanced dataset

After data pre-processing, the ratio between dropout and completers was checked, because it was expected that the dataset would be imbalanced due to a relatively smaller proportion of dropouts compared to completers. This is of importance as an imbalanced dataset causes classification performance problems in machine learning algorithms (52). To deal with this class imbalance, the proven effective synthetic minority oversampling technique (SMOTE) was applied (53). This method over-samples the minority class (i.e., dropout), by artificially creating new samples using the nearest neighbours of the cases, and under-samples the majority class (53).

### 3.3. Model building

For the dropout prediction, a model was developed using Elastic Net Regularization [ENR, R package ‘glmnet’, (54)]. ENR is a combination of ridge regression and lasso regression where alpha is the tuning parameter between ridge regression (alpha = 0) and lasso [alpha = 1; (55)]. Another tuning parameter is the lambda which determines the shrinkage or penalty of the coefficients, the larger its value the stronger the shrinkage (55). The alpha and lambda were determined using 10-fold cross-validation, where the selected alpha was based on the highest area under the curve [AUC; (56)]. After determination of the alpha and lambda, the final model was built.

### 3.4. Model evaluation

The model was evaluated on the initial (imbalanced) dataset using three different measures: accuracy, the area under the curve (AUC), and the Brier score. The selected variables in the final model were evaluated for significance and variable importance. The variable importance was calculated using the vip package (57). The accuracy is the percentage of correct dropout predictions ranging from 0% (worst prediction) to 100% (best prediction). The AUC refers to the overall performance of a classifier. When the AUC is 1, predictions are 100% correct, and when the AUC is 0, all predications are incorrect (56). The Brier score can be described as a parameter that measures the accuracy of probabilistic predictions ranging from 0 (best prediction) to 1 [worst prediction; (58)].

## 4. Results

### 4.1. Data pre-processing

The total sample consisted of 111 patients. From that number, 80 individuals (72%) had no missing data and the percentage of missing baseline variables was 2.5 percent. All patients who dropped out prematurely and were assessed after dropping out still fulfilled the diagnostic criteria for PTSD. The number of sessions before dropping out was registered and the median of the number of sessions before dropping out was 4 sessions. The missing values were imputed and the performance of the imputation method was acceptable (NRMSE = 0.15; PFC 0.26).

### 4.2. Imbalanced dataset and dropout rate

The dropout rate in this study was 19%, indicating that the distribution between dropout and completers was unequal, leading to an imbalanced dataset. To balance the dataset, SMOTE was applied. After applying SMOTE, the dropout rate was 43%.

### 4.3. Model building

The final ENR model selected the following variables for dropout risk: suicidality, benzodiazepine medication use at screening, education, gender, sexual abuse, Borderline personality disorder, PTCI Self-Esteem, SIDES, DERS, complex PTSD, BSI, DES, and CAPS-5 scores.

### 4.4. Predictor evaluation

The best ENR model (alpha = 0.25, lambda = 0.18) correctly predicted 81.6% of all individuals (accuracy). For this model, the AUC was 0.85 and the Brier score was 0.31, indicating sufficient predictions. The sensitivity and specificity are 75 and 87%, respectively. Predictors were evaluated based on both significance and variable importance. Based on significance (i.e., value of p lower than 0.05), the main variables for risk of dropout in the model were gender, education, suicidality, score on the DERS, score on the BSI and benzodiazepine medication use at screening. The (i.e., an importance score greater than or close to 1), the main variables of the model were suicidality, education, and benzodiazepine use at screening (for a total overview of the variable importance, see Table 4). More specifically, low education level, presence of suicidal thoughts, and not using benzodiazepine medication during screening were most strongly related to dropout risk according to both significance and variable importance. Given our focus on prediction, we will further focus on these three variables with the highest variable importance score and not on all significant variables.

**Table 4:** Variable importance indicators.

Variable	Importance
Suicidality	1.22
Benzodiazepine medication use at screening	1.14
Education	0.97
Complex PTSD	0.89
Gender	0.68
SIDES	0.37
DERS	0.35
Sexual Abuse	0.34
PTCI Self-Esteem	0.32
Borderline personality disorder	0.26
BSI	0.24
DES	0.10
CAPS-5	0.07

## 5. Discussion

The purpose of the present study was to identify patients with PTSD related to childhood abuse who are at risk of dropping out of treatment. Therefore, we applied machine learning techniques to analyze all available pre-treatment variables from a recent RCT. The best model was able to correctly predict dropout in 81.6% of the individuals. We consider our research endeavor to be a promising approach for identifying individuals at risk of dropout and, to this end, could be considered a first step in developing models for predicting patient groups that are at an elevated risk of dropout. This makes it possible to develop interventions that support these patients during treatment and prevent them from dropping out; for example, extra nursing support or treatment in clinical settings.

Our model evaluation indicated that the within-sample predictions were accurate based on three different measures (accuracy, AUC, and Brier score). To this end, the combination of pretreatment suicidality, low education, and non-use of benzodiazepines was found to be a risk factor for dropout. Interestingly, from their meta-analysis Guina et al. (59) concluded that the use of benzodiazepines is contraindicated for the treatment of PTSD. They found that the use of benzodiazepines is associated with poor treatment outcomes. To this end, the lack of experienced treatment benefits and the inability to use benzodiazepines as missed opportunities to ease and regulate feelings of tension may explain why patients discontinued therapy. Also, regarding the other predictors, it is conceivable that this could have prompt the patient to discontinue treatment early. This holds true, for instance, if one conceives suicidality as an indicator of feelings of entrapment or hopelessness, and a low level of education as a possible indicator of higher trait impulsivity or low frustration tolerance. However, before speculating too much on how these factors together might explain the increased risk of treatment dropout, more research is needed to first try and replicate these results and rule out the possibility of chance. Our method is the first step toward developing personalized medicine to prevent dropouts from PTSD treatment. Our findings further suggest that one of the ways to reduce dropout rates may be to intensify trauma treatments, as most patients in this study dropped out in an early stage (between the third and fourth treatment sessions) and other studies found that a more intensive PTSD treatment can lead to a lower dropout rate [for example, (60, 61)]. Clearly, replication studies are needed to provide reliable evidence for out-of-sample prediction accuracy for use in clinical practice.

The first strength of this study is that, to our knowledge, this is the first study to analyze predictors of dropout among patients with PTSD related to childhood abuse using machine learning, which has the advantage that it takes into account all possible pre-treatment variables. A second strength is that we handled an imbalanced dataset by applying the proven effective synthetic minority oversampling technique [SMOTE; (53)], which provides more opportunities to compare dropouts with completers, as in most studies, there is a large difference between the sample sizes of both groups. As in any study, some limitations of this study need to be noted. The first limitation is that the study sample was quite small, thereby limiting the possibility of distinguishing between the two treatment conditions (62). Future studies should focus on examining patient characteristics that might predict dropout by machine learning, using larger patient samples and including more types of trauma-focused therapies. A second limitation is the missing data for which we had to impute the data, leading to less reliable outcomes. However, the performance of our imputation method proved reliable (53), which was verified in this study. Third, we were not able to externally validate the data on an independent dataset to answer the question of whether this model leads to significant effects in new samples (63, 64). Before these outcomes are confirmed by external validation, the results cannot be generalized to clinical practice. Although we attempted to overcome this using 10-fold cross-validation, it is unknown how this model will perform on a truly independent dataset. For example, Isaksson et al. (65) argued that cross-validation was unreliable for classifying small samples.

In conclusion, this study identified a combination of variables predicting the dropout rate of patients with PTSD due to childhood abuse in trauma-focused treatments. A challenging task for future research is to examine whether these results can be replicated in larger patient samples. Another challenge is to examine these potential dropout predictors in a more profound way; although our study may help identify patients who are at risk of dropping out of therapy, the results do not reveal the mechanisms that explain the elevated risk. Experimental studies are required to elucidate the exact mechanisms involved, which could be fundamental for future preventive interventions. Developments in the field of machine learning are moving rapidly, and for follow-up research, it may be interesting to look at other models that use tidy modeling.

## Data availability statement

The raw data supporting the conclusions of this article will be made available by the authors, without undue reservation.

## Ethics statement

The studies involving human participants were reviewed and approved by Medical Ethics Committee Twente. The patients/participants provided their written informed consent to participate in this study.

## Author contributions

SBre, NV, SBro, RH, AJ, and MD have made substantial contributions to the design of this study. NV was responsible for the implementation of this study and for the inclusion process and the data collection. AJ supervised the therapists involved in the study. SBre performed the analyses under supervision of SBro. SBre and NV drafted the paper under supervision of SBro, MD, RH, and AJ. All authors contributed to the article and approved the submitted version.

## Funding

Funding for this study was provided by Stichting tot Steun VCVGZ, Dimence Mental Health Care, and the Dutch EMDR association.

## Conflict of interest

AJ receives income from published books on EMDR therapy and training of postdoctoral professionals in this method.

The remaining authors declare that the research was conducted in the absence of any commercial or financial relationships that could be construed as a potential conflict of interest.

## Publisher’s note

All claims expressed in this article are solely those of the authors and do not necessarily represent those of their affiliated organizations, or those of the publisher, the editors and the reviewers. Any product that may be evaluated in this article, or claim that may be made by its manufacturer, is not guaranteed or endorsed by the publisher.

## Footnotes

^2^NTR5991.

^3^56641.044.16 CCMO.
