# Supplementary material for: Predictors of treatment dropout in patients with posttraumatic stress disorder due to childhood abuse
Source: Front Psychiatry. 2023 Aug 4;14:1194669. doi: 10.3389/fpsyt.2023.1194669 (PMC10436563; doi:10.3389/fpsyt.2023.1194669)
Supplement: Supplementary file 1 [file Data_Sheet_1.docx]

**Supplemental Table 1**

| Reasons for dropout given by patients | Dropout n = 23 |
| --- | --- |
| Unknown | 10 |
| Severe physical complains | 3 |
| Problems in living condition or family | 3 |
| Exacerbation of psychiatric complains | 2 |
| Quitting medication | 1 |
| Too fast decline of the PTSD symptoms | 1 |
| Drug abuse (not able to stop during treatment) | 1 |
| Unsatisfied about the treatment | 1 |
| Treatment is too difficult | 1 |

**Supplemental R code**

R code Elastic Net Regularization

# Convert dataset to a matrix

dataset_x <- dataset %>% dplyr::select(-dropout)

dataset_y <- dataset %>% dplyr::select(dropout)

dataset_y2 <- dataset_y

X_dataset <- data.matrix(dataset_x)

Y_dataset <- data.matrix(dataset_y)

# ENR

set.seed(2)

allAlpha <- NULL

nAlphaIterations <- 25

nLambaIterations <- 1000

a <- seq(0, 1, 0.05)

ptm <- proc.time()

############ Tuning the alpha ############

for(j in 1:length(a)){

for (i in 1:nAlphaIterations){

set.seed(i)

cv <- cv.glmnet(X_dataset, Y_dataset, nfold = 10, family = "binomial",type.measure = "class", paralle = F, alpha = a[j])

currAlpha <- data.frame(

MSE = cv$cvm[cv$lambda == cv$lambda.1se],

lambda.1se = cv$lambda.1se,

alpha = a[j],

seed = i)

allAlpha <- rbind(allAlpha, currAlpha) # all current alphas combined

}

print(j)

}

proc.time() - ptm

aggregate(MSE~ alpha, allAlpha, mean)

plot(aggregate(MSE~ alpha, allAlpha, mean))

alphaMin <- aggregate(MSE~ alpha, allAlpha, mean)[aggregate(MSE~ alpha, allAlpha, mean)$MSE == min(aggregate(MSE~ alpha, allAlpha, mean)$MSE) ,"alpha"]

############# Tuning the lambda ############

currentLambda <- NULL

allLambdaIteration <- NULL

nLambaIterations <- 1000

ptm <- proc.time()

for (i in 1:nLambaIterations){

currentLambda <-cv.glmnet(data.matrix(X_dataset), Y_dataset, nfold = 10, type.measure = "class", alpha = alphaMin)

allLambdaIteration <-

rbind(allLambdaIteration,

data.frame(lambdaMin = currentLambda$lambda.min,

lambda1SE = currentLambda$lambda.1se))

print(i)

}

proc.time() - ptm

LambdaMin <- mean(allLambdaIteration$lambdaMin)

set.seed(2)

md3 <- glmnet(X_dataset, Y_dataset, family = "binomial", lambda = LambdaMin, alpha = alphaMin)
